# Seasonal and diel movement patterns of brown bears in a population in southeastern Europe

**DOI:** 10.1002/ece3.8267

**Published:** 2021-10-28

**Authors:** Neda Bogdanović, Anne G. Hertel, Andreas Zedrosser, Milan Paunović, Milan Plećaš, Duško Ćirović

**Affiliations:** ^1^ Faculty of Biology University of Belgrade Belgrade Serbia; ^2^ Behavioral Ecology Department of Biology Ludwig‐Maximilians University of Munich Planegg‐Martinsried Germany; ^3^ Department of Natural Sciences and Environmental Health Faculty of Technology, Natural Sciences and Maritime Sciences University of South‐Eastern Norway Bø i Telemark Norway; ^4^ Department of Integrative Biology Institute of Wildlife Biology and Game Management University of Natural Resources and Applied Life Sciences Vienna Austria; ^5^ Natural History Museum Belgrade Serbia

**Keywords:** brown bear, movement patterns, Serbia, *Ursus arctos*

## Abstract

Most animals concentrate their movement into certain hours of the day depending on drivers such as photoperiod, ambient temperature, inter‐ or intraspecific competition, and predation risk. The main activity periods of many mammal species, especially in human‐dominated landscapes, are commonly set at dusk, dawn, and during nighttime hours. Large carnivores, such as brown bears, often display great flexibility in diel movement patterns throughout their range, and even within populations, striking between individual differences in movement have been demonstrated. Here, we evaluated how seasonality and reproductive class affected diel movement patterns of brown bears of the Dinaric‐Pindos and Carpathian bear populations in Serbia. We analyzed the movement distances and general probability of movement of 13 brown bears (8 males and 5 females) equipped with GPS collars and monitored over 1–3 years. Our analyses revealed that movement distances and probability of bear movement differed between seasons (mating versus hyperphagia) and reproductive classes. Adult males, solitary females, and subadult males showed a crepuscular movement pattern. Compared with other reproductive classes, females with offspring were moving significantly less during crepuscular hours and during the night, particularly during the mating season, suggesting temporal niche partitioning among different reproductive classes. Adult males, solitary females, and in particular subadult males traveled greater hourly distances during the mating season in May‐June than the hyperphagia in July–October. Subadult males significantly decreased their movement from the mating season to hyperphagia, whereas females with offspring exhibited an opposite pattern with almost doubling their movement from the mating to hyperphagia season. Our results provide insights into how seasonality and reproductive class drive intrapopulation differences in movement distances and probability of movement in a recovering, to date little studied, brown bear population in southeastern Europe.

## Graphical abstract

Large carnivores, such as brown bears, often display great flexibility in diel movement patterns throughout their range, and even within populations, striking between individual differences in movement have been demonstrated. Here, we evaluated how seasonality and reproductive class affected diel movement patterns of brown bears of the Dinaric‐Pindos and Carpathian bear populations in Serbia. We analyzed the movement distances and general probability of movement of 13 brown bears (8 males and 5 females) equipped with GPS‐collars and monitored over 1–3 years. Our results provide insights how seasonality and reproductive class drive intra‐population differences in movement distances and probability of movement in a recovering, to‐date little studied, brown bear population in south‐eastern Europe.

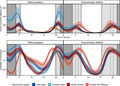

## 1 INTRODUCTION

The diel and seasonal movement patterns of mammals are shaped by a suite of environmental drivers, among them photoperiod (Nielsen, 1983), temperature (Pigeon et al., 2016; Seryodkin et al., 2013), food availability (Heurich et al., 2014; Klinka & Reimchen, 2009), and inter‐ and intraspecific competition (Monterroso et al., 2013), although seasonal variation in human activity (Gaynor et al., 2018; Marchand et al., 2014) can further modify these patterns. The rapid growth of human populations has forced many wild animals to share their living space with humans, in the so‐called human‐dominated landscapes (Gaynor et al., 2018; Zarzo‐Arias et al., 2018). Under such conditions, the possibility of human–wildlife encounters increases significantly, which is why many animals, in order to avoid potential encounters, shift their movement to times when human activity is low (Brook et al., 2012; Gaynor et al., 2018; Ordiz et al., 2014). For carnivores, significant shifts in diel activity toward the dark and crepuscular hours of the day have been observed in human‐dominated landscapes (Gaynor et al., 2018; Wu et al., 2018), which is considered to be a consequence of anthropogenic stress (Seryodkin et al., 2013).

The brown bear (*Ursus arctos*) is a large carnivore that inhabits human‐dominated landscapes in Europe (Chapron et al., 2014; Swenson et al., 1999; Zedrosser et al., 2001). Bears show natural variation in movement patterns over the course of the year as a result of their life history, which includes three important stages, that is, mating, hyperphagia, and hibernation (Swenson et al., 2000). Additionally, intraspecific interactions (both attraction and avoidance) are important factors for shaping bear behavior, leading to variations between different reproductive classes (Kaczensky et al., 2006; Lewis & Rachlow, 2011). During the mating season, which usually occurs in the late spring/early summer, movement patterns of adult bears are predominantly shaped by reproductive behavior, that is, the search and courting of partners (Dahle & Swenson, 2003; Steyaert et al., 2012). Adult females with dependent cubs of the year try to avoid adult males during this time period to avoid infanticide (Steyaert et al., 2013, 2014; Swenson et al., 2003), whereas subadults modify their behavior as a result of natal dispersal (Zedrosser et al., 2007). During the hyperphagia season in summer and autumn, movement of all reproductive classes is mostly driven by food search to increase adipose tissue in preparation for hibernation. Although natural food resources are often widely dispersed (Hertel, Steyaert, et al., 2016), artificial feeding sites can provide a clumped, high‐calorie food sources which can alter bear movement patterns (Kavčič et al., 2013; Selva et al., 2017; Ziegltrum & Nolte, 1997). Also, humans greatly affect bear behavior and life history (Hertel, Zedrosser, et al., 2016; Ordiz et al., 2014; Van de Walle et al., 2018; Zedrosser et al., 2011), and bears generally try to avoid humans on a spatio‐temporal scale, that is, bears move mostly during night and crepuscular hours when human activity on the landscape is lower (Kaczensky et al., 2006; Ordiz et al., 2014; Parres et al., 2020; Roth, 1980; Roth & Huber, 1986). Therefore, sustainable bear conservation and management must take into consideration the natural patterns of bear movement as well as the behavioral responses to human disturbance (Hertel et al., 2017; Tuomainen & Candolin, 2011; Zarzo‐Arias et al., 2018).

Brown bears in Serbia are at the interface of the Dinaric‐Pindos and the Carpathian populations and, thus, represent a potential connection for genetic exchange between these two large populations in southeastern Europe (Ćirović et al., 2015). This makes bears in Serbia of particular conservation concern, which in combination with increasing human impact on brown bear habitats and their strictly protected status (Ćirović & Paunović, 2018), requires the application of well‐planned conservation actions. Here, we carry out a systematic analysis of bear diel movement patterns in Serbia, with the goal to improve future bear management and conservation.

The main aim of this study was to evaluate the differences in seasonal and diel movement patterns for different reproductive classes of brown bears in a human‐dominated landscape and area of great conservation concern. Based on existing literature of diel movement patterns of brown bears in human‐dominated landscapes (Ćirović et al., 2015; Hertel et al., 2017; Kaczensky et al., 2006; Parres et al., 2020), we predicted (i) that bears would follow a bimodal movement pattern with periods of high movement during crepuscular hours and that (ii) bears would move over longer distances during the mating than during the hyperphagia season. We further predicted that (iii) dispersing subadult males would travel longer distances during the mating season than adults and females with dependent offspring and that (iv) differences in movement patterns between reproductive classes would be less pronounced during the hyperphagia than during the mating season.

## 2 MATERIALS AND METHODS

### 2.1 Study areas and bear capture

**FIGURE 1:**
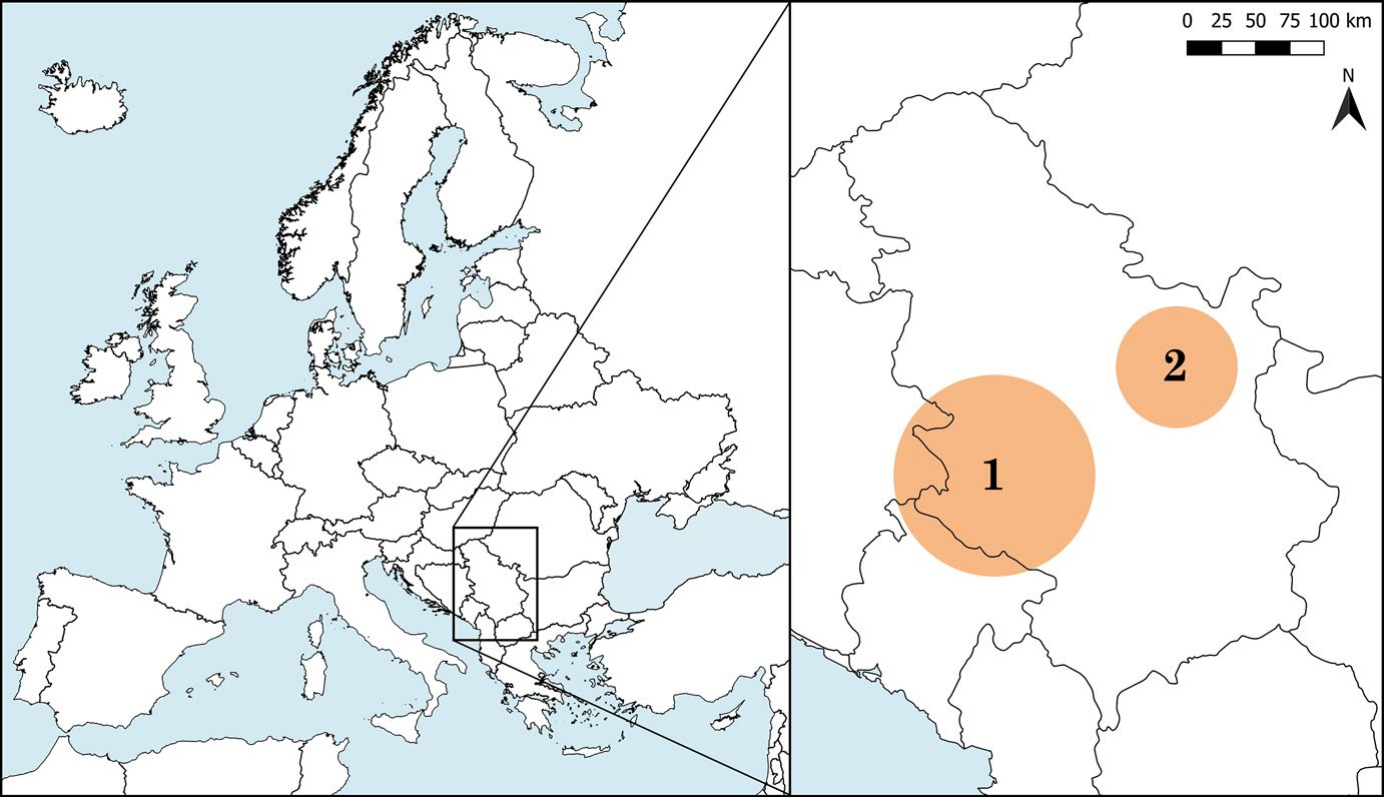
Location of study areas for seasonal and diel activity patterns of brown bears in Serbia, 2007–2019; (1) Stari Vlah‐Raška Mountain Range, (2) Južni Kučaj Mountain

Bears were monitored in two study areas in Serbia, the Stari Vlah‐Raška Mountain Range (~43°50′, 19°27′), which is part of the Dinaric‐Alps in southwestern Serbia, as well as on Južni Kučaj Mountain (~44°05′, 21°50′), which is part of the Carpathian Mountain Range in eastern Serbia (Figure 1). There is no connection between these two populations (Ćirović & Paunović, 2018). The Stari Vlah‐Raška Mountain Range has altitudes ranging from 750 to 1500 m above sea level (Pavlović et al., 2017), and ~35% of the area (240,000 ha) is covered with dense forests dominated by silver fir (*Abies alba*), Norway spruce (*Picea abies*), and beech (*Fagus* spp.). The rest of the landscape is covered with agricultural land, such as pastures, meadows, and orchards. (Pavlović & Živković, 2003). The bear population in southwestern Serbia, with an estimated population size of 60 ± 10 bears and a slightly increasing population trend, is considered part of the large Dinaric‐Pindos population (Chapron et al., 2014; Kaczensky et al., 2013). Južni Kučaj Mountain (max. elevation 1284 m) is mostly covered by beech and beech–coniferous forests (72% of the area), and agricultural land covers the remaining 29%. The bear population in eastern Serbia is very small (~6 individuals) but is considered part of the large Carpathian bear population (Chapron et al., 2014; Kaczensky et al., 2013). The only monitored bear from this population was a female that had been translocated from the Dinaric‐Pindos population in western Serbia in 2007. Bear populations in both areas have access to a network of supplementary feeding sites for ungulates as well as diversionary feeding sites used to prevent bears from searching for food near humans.

We captured bears using Aldrich foothold snares (Johnson & Pelton, 1980) in the time period 2007–2019. Bears were chemically immobilized with an intramuscular injection of 3 ml tiletamine–zolazepam (Zoletil 100; Virbac, Prague, Czech Republic; initial vol 10 mg/kg) using a dart gun (Dan‐Inject^®^, Børkop, Denmark). Standard body measurements were taken at each capture, and age was estimated based on body mass and size, as well as tooth wear (Jonkel, 1993; Karamanlidis et al., 2015). Each bear was equipped with a GPS collar with GSM download (GPS Plus; Vectronic Aerospace GmbH, Berlin, Germany) and a timer‐controlled drop‐off system. Bears were released at the trap site (Ćirović et al., 2015), with the exception of one adult female, which was translocated for management reasons from the Stari Vlah‐Raška Range to Južni Kučaj in 2007. All GPS collars were scheduled to record a location every 1 h (24 positions/day). The GPS relocation success rate during the active period ranged from 65 to 97% (mean: 84%). Permit for the capture and handling brown bears was provided by the Ministry of Environmental Protection (license number: 353‐01‐1053/2019‐04).

### 2.2 GPS data, intensity, and probability of movement

We analyzed diel movement patterns for 8 males and 5 females followed over 1–3 years (i.e., “bearyear”—each year during which a bear was monitored) (Table 1). GPS‐collared brown bears were grouped according to their sex and age into subadults (<5 years) and adults (≥5 years) (Dahle & Swenson, 2003; Elfström & Swenson, 2009). Females were further distinguished by their reproductive status as being accompanied by offspring or being solitary. This led to four categories: adult males (nBearyear = 8), subadult males (nBearyear = 6), solitary females (nBearyear = 8), and females with dependent offspring (nBearyear = 4) (Hertel et al., 2017; Ordiz et al., 2007; Steyaert et al., 2013; Table 1).

**TABLE 1:** Data used in the analysis of movement patterns of brown bears in Serbia, 2007–2019

Reproductive class	Bear ID	Study area	Observation days used in analysis	Number of bearyears
Adult males	Batica	Stari Vlah	129	2
	Dobrivoje	Stari Vlah	181	1
	Ogi	Stari Vlah	366	2
	Rača	Stari Vlah	260	2
Subadult males	Andrej	Stari Vlah	96	2
	Miloje	Stari Vlah	183	1
	Miloš–2019 (adult)	Stari Vlah	372	3
	Zoran	Stari Vlah	68	1
Solitary females	Milica	Južni Kučaj	374	3
	Sonja	Stari Vlah	332	2
Females with dependent offspring	Flekica	Stari Vlah	336	2
	Medena	Stari Vlah	219	2
	Slobodanka	Stari Vlah	464	3

Bears in southeastern Europe hibernate approximately from the end of November until the end of March (Kaczensky et al., 2006). We divided the active period of the year (from May 1 until October 31, i.e., outside of the hibernation period) into two distinct seasons: the mating season, which lasts 2 months in spring and early summer (defined here as May 1–June 30), and hyperphagia season, which occurs after mating is completed until hibernation in autumn (defined as July 1–October 31) (Ciucci et al., 2014; Steyaert et al., 2013). We excluded the months of April and November from the analysis due to the very low number of locations (some bears may not have emerged from hibernation or have already entered the den). We extracted sunrise, sunset, and day length for every day in the mating and hyperphagia seasons for Central European Time (UTC + 1) and Central European Summer Time (UTC + 2) with the library *maptools* (Bivand & Lewin‐Koh, 2014) using N 43°82" and E 19°72" (the village Tripkova, Zlatibor Mountain, Serbia) as reference coordinates. We further extracted civil dusk and dawn, that is, the time of day when the sun is between 6 and 0 degrees below the horizon (Ensing et al., 2014). The crepuscular hours of the day were defined as the time period between civil dawn until sunrise (morning twilight) and from sunset until the end of civil dusk (evening twilight), and periods between sunrise and sunset and civil dusk and dawn were defined as day and night, respectively.

### 2.3 Movement metrics

To describe bear movement patterns, we calculated two complementary metrics: (a) hourly movement distance, that is, meters/h and (b) probability of movement, a binary metric of whether a bear moved (>50 m) or was stationary (<50 m) during a given hour of a day. We first calculated hourly movement distance as a measure of intensity of movement, that is, how much do bears move during any given hour of 24‐h period. We then constructed regular movement trajectories for every bearyear, using the library *adehabitatLT* (Calenge, 2006). Hourly movement distances were extracted from the trajectories as the Euclidean distance between two successive hourly locations. To avoid erroneous distance calculations (i.e., displacements over two hours or longer), all missing locations were set to NA, resulting in the removal of distance calculations one hour before and after a missing location (Hertel, Steyaert, et al., 2016). We further calculated the probability of movement by categorizing hourly movement distances into moving and stationary positions. Bears were considered stationary when the distance between two successive hourly locations was ≤50 m (coded as 0’s), that is, two times the average GPS positional error (25 m) (Ćirović et al., 2015), whereas all movement distances >50 m (coded as 1’s) were defined as moving positions.

### 2.4 Statistical methods

We used generalized additive mixed models (GAMMs) to test for temporal trends in the movement distance and probability of movement during the course of 24 h by fitting a cyclic cubic spline over hour of day. In addition, we tested for temporal differences among reproductive classes (adult males, subadult males, solitary females, and females with dependent offspring) and seasons (mating and hyperphagia).

Hourly movement distance (in meters), that is, intensity of movement, was modeled as GAMM with a Gaussian distribution using the mgcv package (Wood, 2011). We used diagnostic plots to validate that the distribution of the residuals was normal and homogeneous. To improve model fit, we refitted models with a log‐transformed response variable. We back‐transformed model predictions to the original scale (meters) for better model interpretation.

Probability of movement was modeled as a binary response variable: moving (1) versus stationary (0) hourly intervals in a GAMM with a binomial distribution using the R package gamm4 (Wood & Scheipl, 2013). Hence, the model‐predicted ratio between stationary and moving increments at any hour of the day represents a probability of movement (i.e., when bears move versus not move). We controlled for consistent among‐individual differences in movement distance and probability of movement with a random intercept for Bearyear. We fitted two sets of models (Table 2): first, we tested whether diel movement patterns differed among reproductive classes by fitting a cyclic cubic spline over time of day interacting with reproductive class (bearclass model; Table 2). We fit this model separately for the mating and hyperphagia periods. Second, we tested whether diel movement patterns (for each reproductive class separately) differed among two seasons by fitting a cyclic cubic spline over time of day interacting with season (Seasonal model; Table 2). We used “by” function to include an interaction term in all models. Because it was not possible to fit a three‐way interaction (day, season, and reproductive class), we split our analyses into two model sets in order to interpret the contrast both among seasons but also among reproductive classes. We tested models against a simpler model not controlling for variation in the temporal trend among reproductive classes or seasons, respectively (Table 2). We selected the most parsimonious model based on second‐order bias‐corrected Akaike's information criterion (AIC), that is, models with an Akaike weight (AICcw) close to 1 receive most support relative to other candidate models (Tables S1 and S2) (Arnold, 2010). We validated model assumptions (normal distribution of residuals and absence of heteroscedasticity) by plotting residuals against fitted values. We controlled for inherent temporal autocorrelation in the movement data with the use of a spline over hour of the day and confirmed that no unmodeled temporal autocorrelation remained in the model (Figures S1 and S2).

**TABLE 2:** Candidate models to explain the temporal trends in movement distance and probability of movement of brown bears in Serbia, 2007–2019, in relation to reproductive class (adult male, subadult male, solitary female, and female with dependent offspring), that is, bearclass model and season (mating and hyperphagia season), that is, seasonal model

	Explanation
Bearclass model	
Movement distance/probability of movement ~s(hour, by=reproductive class) + reproductive class	s(hour, by = reproductive class) denotes the differences in the movement distance and probability of movement between the reproductive classes at different hours of the day, and “reproductive class” denotes the general differences in movement and probability of movement between the reproductive classes
Movement distance/probability of movement ~s(hour, by = reproductive class)	s(hour, by = reproductive class) denotes the differences in the movement distance and probability of movement between the reproductive classes at different hours of the day
Seasonal model	
Movement distance/probability of movement ~s(hour, by = season) + season	s(hour, by = season) denotes the differences in the movement distance and probability of movement between the seasons at different hours of the day, and “season” denotes the general differences in movement and probability of movement between seasons
Movement distance/probability of movement ~s(hour, by = season)	s(hour, by = season) denotes the differences in the movement distance and probability of movement between the seasons at different hours of the day

Note

Reproductive class = factor with four levels (the adult males, subadult males, single females, females with dependent offspring), season = factor with two levels (mating season, hyperphagia season). The interaction term “by” in the respective first models allows that the effect of hour of the day on the response variable differs between factor levels.

Finally, we used generalized linear mixed models (GLMMs) to contrast differences in the probability of movement (binary response variable with moving versus stationary positions). We fitted principal periods of a day (factor with three levels: day, night, and crepuscular hours) as explanatory variables for each reproductive class (adult males, subadult males, single females, and females with dependent offspring) and season (mating versus hyperphagia) separately, using the package lme4 (Bates et al., 2014). The statistical software R 3.6.1 (R Development Core Team, 2019) was used in all analyses.

## 3 RESULTS

We analyzed movement patterns of 13 brown bears monitored for 1–3 years (a total of 26 monitoring years, i.e., “bearyear”). During the active period of the year (May 1–October 31), individual bears were monitored for a minimum period of 20 days and a maximum of 184 days (mean monitoring period: 132 days).

### 3.1 Movement distance

#### 3.1.1 Bearclass model

Movement distance was affected by the time of day and differed among reproductive classes during both the mating season (ΔAIC = −1767.5, AICcw = 1) and the hyperphagia season (ΔAIC = −904.1, AICcw = 1; Table S1).

**FIGURE 2:**
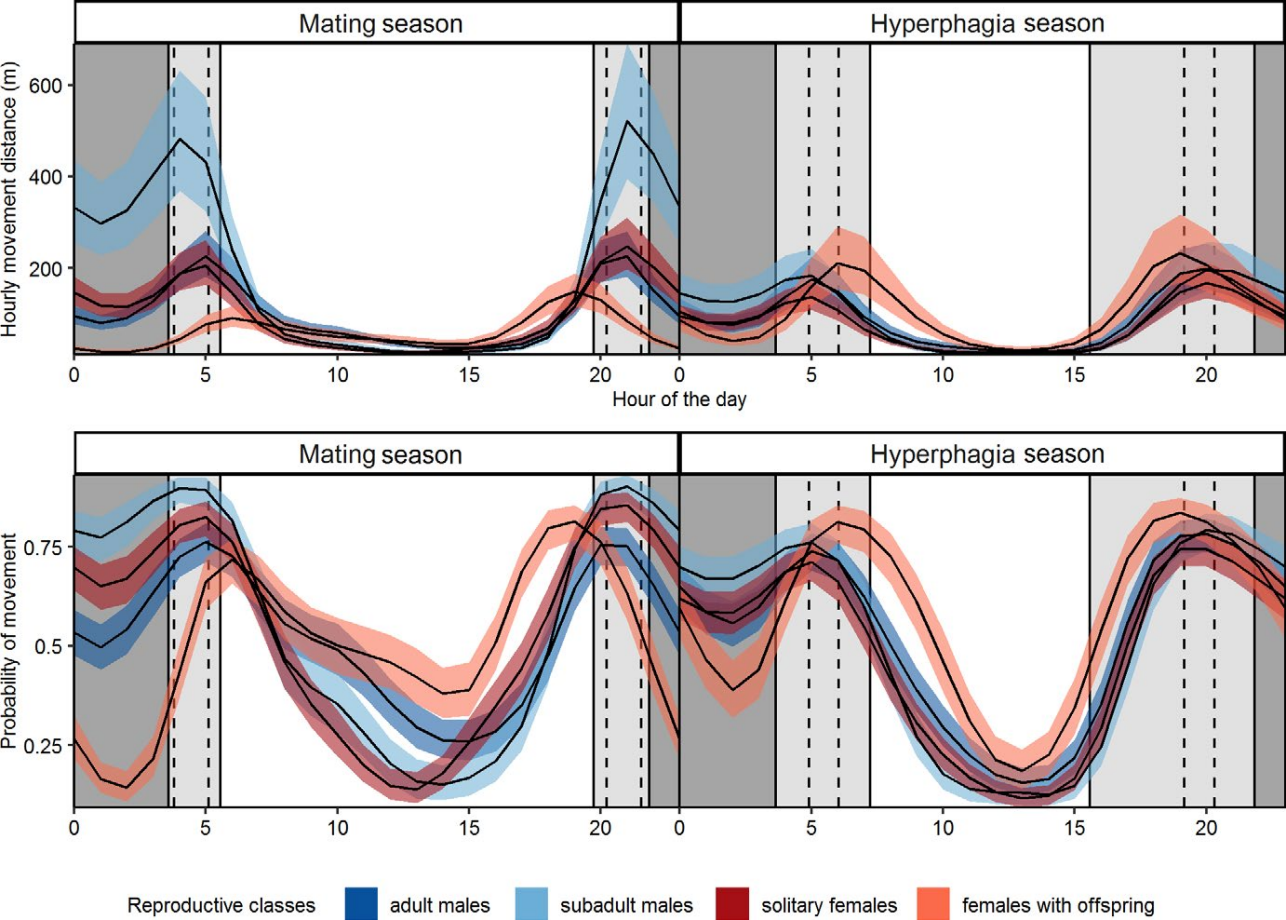
Predicted average hourly movement distances (upper panel) and predicted probability of movement within 24 h (lower panel) of 4 reproductive classes of brown bears (adult male, subadult male, solitary female, female with offspring) per season (mating: left panels, hyperphagia: right panels) in Serbia, 2007–2019. Vertical lines represent dawn, sunrise, sunset, and dusk, dividing a 24‐h period into night (dark grey area), crepuscular hours (light grey area), and day (white area). Solid lines represent the maximum duration of the crepuscular period during both seasons, and dotted lines represent mean value

During the mating season, movement distances for three out of four reproductive classes (adult males, subadult males, solitary females) were longest during the crepuscular hours before sunrise and after sunset and shortest during daytime, reaching their minimum level around noon (Figure 2, upper panel). Subadult males moved the longest hourly distances as compared with all other reproductive classes (~550 m, between 20:00–21:00 and 3:00–4:00; Figure 2 (upper left panel – light blue line); *β* = 4.63; Table 3). Solitary females and adult males moved significantly less during the same time periods (~230 m; Figure 2, upper left panel – red and dark blue lines, respectively; *β*_adult males_ = 4.38 and *β*_single females_ = 4.27; Table 3). On the contrary, females with dependent offspring moved the longest hourly distances after sunrise (~100 m between 5:00–6:00) and before sunset (~180 m between 18:00–19:00; Figure 2 (upper left panel–orange line)) and moved significantly less during crepuscular hours and during night than the other reproductive classes (Figure 2, upper left panel – orange line).

**TABLE 3:** Coefficients and standard errors (*β* ± SE) for the explanatory variable as well as the significance of the smoothing terms (edf, Chi.sq/*F*) obtained in the most parsimonious bearclass model predicting movement distance and probability of movement in the mating and hyperphagia period in relation to reproductive class of brown bears in Serbia, 2007–2019

		Mating period			Hyperphagia period	
Movement distance		***β***	**SE**		***β***	**SE**
	(Intercept)	4.63	0.12	(Intercept)	4.22	0.13
	Adult males	−0.25	0.15	Adult males	0.05	0.17
	Single females	−0.36	0.15	Single females	−0.17	0.17
	Females with offspring	−0.71	0.16	Females with offspring	0.20	0.20
		**edf**	***F***		**edf**	***F***
	Hour:subadult males	7.72	301.3	Hour:subadult males	7.74	413
	Hour:adult males	7.72	114.2	Hour:adult males	7.83	371
	Hour:single females	7.59	175.9	Hour:single females	7.84	604.2
	Hour:females with offspring	7.65	80.6	Hour:females with offspring	7.83	235.2
Probability of movement		***β***	**SE**		***β***	**SE**
	(Intercept)	0.45	0.14	(Intercept)	0.03	0.12
	Adult males	−0.31	0.17	Adult males	0.08	0.16
	Single females	−0.22	0.17	Single females	−0.12	0.16
	Females with offspring	−0.47	0.18	Females with offspring	0.32	0.19
		**edf**	**Chi.sq**		**edf**	**Chi.sq**
	Hour:subadult males	7.59	998.8	Hour:subadult males	7.59	1828
	Hour:adult males	7.57	490.3	Hour:adult males	7.72	1893
	Hour:single females	7.56	900.2	Hour:single females	7.77	2789
	Hour:females with offspring	7.74	643.3	Hour:females with offspring	7.77	1303

In comparison to the mating season, the discrepancy in movement distances between reproductive classes was significantly less pronounced during the hyperphagia season. Movement distances were longest during crepuscular hours and night for all reproductive classes, with females with dependent offspring showing a later peak in the morning and an earlier peak in the evening (Figure 2, upper right panel). During the hyperphagia season, females with dependent offspring traveled slightly longer hourly distances than the other three reproductive classes (~220 m between 18:00–19:00 and 5:00–6:00; Figure 2, upper right panel: orange line; *β*_females with offspring_ = 4.44; Table 3), whereas solitary females moved the least during the same period (~150 m; Figure 2 (upper right panel ‐ red line); *β*_single females_ = 4.05; Table 3).

#### 3.1.2 Seasonal model

Movement distance in relation to time of the day differed among seasons for adult males (ΔAIC = −168.5, AICcw = 1), subadult males (ΔAIC = −282.3, AICcw = 1), solitary females (ΔAIC = −117.6, AICcw = 1), and females with offspring (ΔAIC = −619.7, AICcw = 1) (Table S2).

**FIGURE 3:**
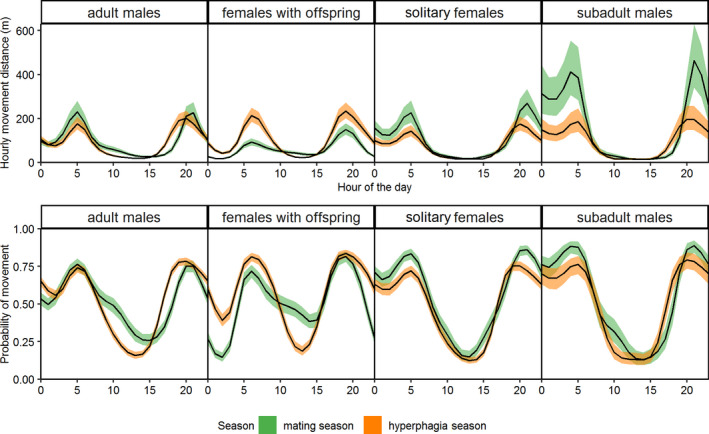
Predicted average hourly movement distances (upper panel) and predicted probability of movement within 24‐hour (lower panel) of brown bear reproductive classes (adult male, subadult male, solitary female, female with dependent offspring) during the mating (green line) and hyperphagia (orange line) seasons in Serbia, 2007–2019

Adult males, solitary females, and in particular subadult males traveled greater distances during the mating season than during the hyperphagia season (Table 4, Figure 3, upper panel). Subadult males decreased their hourly movement distance from 463 m (between 20:00 and 21:00) in the mating season to 196 m during the same time period in the hyperphagia season (Figure 3, upper panel; *β*_mating period_ = 4.49 and *β*_hyperphagia period_ = 4.20; Table 4). Females with dependent offspring traveled shorter distances, that is, 92 m (between 5:00 and 6:00) during the mating season and increased their movement to 214 m for the same time periods during hyperphagia (Figure 3, upper panel; *β*_mating period_ = 3.92 and *β*_hyperphagia period_ = 4.42; Table 4).

**TABLE 4:** Coefficients and standard errors (*β* ± SE) for explanatory variable as well as significance of the smoothing terms (edf, Chi.sq/*F*) obtained in the most parsimonious seasonal model predicting movement distance and probability of movement for 4 reproductive classes of brown bears (adult male, subadult male, solitary female, female with dependent offspring) in relation to seasonal variation (mating and hyperphagia) in Serbia, 2007–2019

		Adult males		Females with offspring		Single females		Subadult males	
Movement distance		***β***	**SE**	***β***	**SE**	***β***	**SE**	***β***	**SE**
	(Intercept)	4.23	0.07	4.42	0.06	4.09	0.08	4.20	0.13
	Mating	0.12	0.03	−0.50	0.03	0.28	0.03	0.29	0.04
		**edf**	***F***	**edf**	***F***	**edf**	***F***	**edf**	***F***
	Hour:hyperphagia	7.81	322.8	7.84	257.9	7.85	616	8.76	353.4
	Hour:mating	7.69	106.9	7.69	94.6	7.64	193.4	8.75	274.4
Probability of movement		***β***	**SE**	***β***	**SE**	***β***	**SE**	***β***	**SE**
	(Intercept)	0.11	0.05	0.35	0.06	−0.03	0.07	0.01	0.15
	Mating	0.02	0.04	−0.37	0.04	0.35	0.04	0.26	0.06
		**edf**	**Chi.sq**	**edf**	**Chi.sq**	**edf**	**Chi.sq**	**edf**	**Chi.sq**
	Hour:hyperphagia	7.72	1890.7	7.77	1300.3	7.76	2768.5	7.59	1830
	Hour:mating	7.57	493.7	7.74	640.7	7.56	902.7	7.59	1004

### 3.2 Probability of movement

#### 3.2.1 Bearclass model

The probability of movement was affected by the time of day and differed among reproductive classes during the mating (ΔAIC = −1389.7, AICcw = 1) and hyperphagia periods (ΔAIC = −581.4, AICcw = 1; Table S1). During the mating season, females with dependent offspring showed the highest probability for movement during day (59%) and crepuscular hours (57%) (*β*_day_ =0.36 and *β*_crepuscular_ = 0.26; Table 5), but their nocturnal movement level was low (24% probability of movement; *β*_night_ = −1.15; Table 5). Movement of adult males, subadult males, and solitary females occurred predominantly during crepuscular hours (75%, 93%, and 84%, respectively) and during night (56%, 81%, and 70%, respectively; Table 5).

**TABLE 5:** Coefficients and standard errors (*β* ± SE) obtained in the generalized linear mixed model (GLMMs) predicting probability of movement for 4 reproductive classes of brown bears (adult male, subadult male, solitary female, female with dependent offspring) in relation to period of the day (day, night, crepuscular) during the mating and hyperphagia seasons in Serbia, 2007–2019

	Adult males				Females with offspring			
	Mating period		Hyperphagia period		Mating period		Hyperphagia period	
	*β*	SE	*β*	SE	*β*	SE	*β*	SE
(Intercept:day)	−0.08	0.06	−0.39	0.06	0.36	0.10	0.18	0.06
Night	0.31	0.06	0.94	0.04	−1.51	0.07	0.01	0.05
Twilight	1.18	0.10	2.08	0.08	−0.10	0.09	1.56	0.09

	Solitary females				Subadult males			
	Mating period		Hyperphagia period		Mating period		Hyperphagia period	
	*β*	SE	*β*	SE	*β*	SE	*β*	SE
(Intercept:day)	−0.20	0.16	−0.61	0.11	−0.29	0.16	−0.76	0.17
Night	1.07	0.07	1.09	0.03	1.77	0.09	1.63	0.05
Twilight	1.83	0.19	1.94	0.06	2.83	0.19	2.33	0.10

During the hyperphagia season, probability of movement for all reproductive classes was highest during the crepuscular hours (Table 5). Adult males, subadult males, and solitary females were twice as likely to move during crepuscular hours (84%, 83%, 79%; Table 5) as during the day (40%, 32%, 35%; Table 5).

#### 3.2.2 Seasonal model

The probability of movement within 24 hours differed between seasons for adult males (ΔAIC = −186.5, AICcw = 1), subadult males (ΔAIC = −93.2, AICcw = 1), solitary females (ΔAIC = −85.6, AICcw = 1), and females with dependent offspring (ΔAIC = −408.6, AICcw = 1; Table S2). Adult males, subadult males and solitary females showed a slightly increased probability of movement during the mating season, compared with the hyperphagia season, whereas females with dependent offspring showed the opposite pattern, that is, slightly higher probability of movement level during hyperphagia (Table 4; Figure 3, lower panel). Females with dependent offspring change their probability of movement between mating (highest movement probability during daylight hours – 59%; *β*_day_ = 0.36; Table 5) and hyperphagia seasons (highest probability during crepuscular hours – 85%; *β*_crepuscular_ = 1.74; Table 5). In addition, they almost doubled their probability of movement during night and crepuscular hours in the hyperphagia season (55% and 85%), compared with mating season (24% and 57%, respectively; Table 5).

## 4 DISCUSSION

Our results generally support our predictions that diel movement patterns of brown bears in Serbia varied among different reproductive classes and between seasons. Based on our results, bears in our study area showed greatest movement rates during crepuscular and night hours, and lowest movement rates during diurnal time periods (support prediction i). In more detail, subadult males were mostly crepuscular, adult males, and solitary females were moving both during night and crepuscular hours with no pronounced resting period during the night, whereas females with offspring were moving more during daylight and crepuscular hours than other reproductive classes. This corresponds well with the findings of Moe et al. (2007) for Scandinavian female bears and with findings of Kaczensky et al. (2006) for adult brown bears in Slovenia and Croatia. Contrary to findings of Kaczensky et al. (2006) and Parres et al. (2020) that subadult bears are significantly more active during the day, and we found that subadult males in our study areas exhibited mostly crepuscular and nocturnal movement patterns during both the mating and hyperphagia periods and traveled greater distance than any other reproductive class during the dark hours of the mating season (support prediction) (iii). Nocturnality of younger individuals has also been observed in Scandinavian brown bears (Hertel et al., 2017). This movement pattern probably represents a strategy to avoid encounters with crepuscular adult male bears and may reduce competition for food and space between these two classes.

However, the temporal niche partitioning observed in our study was less pronounced than other areas of the Dinaric‐Pindos population (Kaczensky et al., 2006). This is likely related to the small population sizes in our study areas (~60 individuals in the Dinaric‐Pindos and ~6 individuals in the Carpathian population) compared with Slovenia (~440 individuals) and Croatia (~1000 individuals) (Chapron et al., 2014; Kaczensky et al., 2013), that is, the relatively low population size may result in lower competition and reduced temporal niche partitioning compared with larger populations. In their study, Kaczensky et al. (2006) suggested that subadults are more day active to reduce food competition with nocturnal adults. Thus, the relatively small number of individuals in our study area and the resulting lower probability of encounters may explain why subadult bears are moving at similar times as other bear age and sex classes.

The generally higher movement rates of subadult males were likely related to natal dispersal (Zedrosser et al., 2007). Adult male and solitary female bears showed greater movement rates during the mating season than during the hyperphagia season (support prediction (ii), most likely due to mate search behavior. This assumption is supported by the results obtained by Dahle and Swenson (2003) who showed that both adult males and solitary females significantly decreased their ranges from mating to post‐mating season (which overlaps with our definition of the hyperphagia season). Both classes had movement peaks at similar times of the day (Figure 2). A comparable pattern was also found in adult males and solitary females in the Pyrenean brown bear population during spring, which coincides with the mating season (Parres et al., 2020). Diurnal movement in adult bears in our study areas was very low during both seasons, which corresponds to the results obtained for adult bears in Slovenia and Croatia (Kaczensky et al., 2006).

All reproductive classes, except females with dependent offspring, decreased their movement rates from mating to the hyperphagia season (support prediction ii), with movement occurring mostly during night and crepuscular hours. Several studies have shown that bear activity is negatively affected by human presence (Hertel et al., 2017; Martin et al., 2010; Matthews et al., 2006; Nellemann et al., 2007). Parts of our study area are a popular tourist destination in Serbia, and the resulting human activity may be one of the reasons for the high degree of nocturnal movement of bears during both season; however, no data were available to test this effect.

Numerous previous studies of bear movement and activity patterns emphasize the impact of artificial feeding or baiting stations (intended either for bears and/or other wildlife) on bear movement patterns (Cozzi et al., 2016; Elfström et al., 2014; Fersterer et al., 2000; Jerina et al., 2013; Penteriani et al., 2017; Selva et al., 2017; Steyaert, Kindberg, et al., 2014). Although artificial feeding stations are present in our study area, due to insufficient data regarding their number, locations, and food supplementation frequencies for the entire monitoring period, we cannot evaluate their effect on bear movement rates. When feeding stations are present in a bears home range, we expect a reduced movement rate, in particular during the hyperphagia period because bears stay closer to the feeding stations. Future research should focus on the effect of feeding stations on bear movement patterns.

Females with dependent offspring showed a contrasting movement pattern compared with other reproductive classes, with movement occurring predominantly during daylight (mating season) and crepuscular hours (hyperphagia season). This is in line with other studies showing that females with offspring are more diurnal than other reproductive classes (Kaczensky et al., 2006; Munro et al., 2006; Parres et al., 2020; Rauer et al., 2003; Steyaert, Swenson, et al., 2014). During the mating season, adult males may kill cubs of the year (Steyaert, Swenson, et al., 2014), and females with cubs try to avoid infanticide by shifting their movement into daytime hours (Dahle & Swenson, 2003; Edwards et al., 2013; Steyaert, Swenson, et al., 2014; Wielgus & Bunnell, 1995). Alternatively, diurnal movement may provide easier accessibility to food sources which are occupied by other bears during the night. This assumption is in accordance with findings by Klinka and Reimchen (2002) and Kaczensky et al. (2006), who suggest that diurnal activity of females with offspring can be advantageous in relation to food accessibility and offspring safety. We found a significant increase in movement rates of females with dependent offspring for all periods of the day during hyperphagia, which is possibly related to increased mobility and nutritional needs of offspring.

Our results suggest that movement patterns of adult bears (males and solitary females) during the mating season are strongly influenced by mating behavior, whereas subadults males and females with dependent offspring modify their movement in order to disperse or to reduce infanticide risk. During the hyperphagia season, these behavioral differences in movement distances and probability of movement between reproductive classes disappeared (support prediction iv), and movement seemed mostly driven by food search. In addition, bear movement patterns may be affected by feeding stations and tourism. Additional research is needed to better understand bear movement ecology in areas with supplemental feeding programs and rapidly increasing tourism.

## CONFLICT OF INTEREST

The authors declare no conflict of interest.

## AUTHOR CONTRIBUTIONS

**Neda Bogdanović:** Conceptualization (equal); Data curation (equal); Formal analysis (supporting); Investigation (equal); Methodology (equal); Validation (equal); Writing‐original draft (lead); Writing‐review & editing (equal). **Anne G Hertel:** Conceptualization (equal); Data curation (equal); Formal analysis (lead); Investigation (equal); Methodology (equal); Supervision (equal); Validation (equal); Writing‐original draft (supporting); Writing‐review & editing (equal). **Andreas Zedrosser:** Conceptualization (equal); Formal analysis (supporting); Funding acquisition (supporting); Investigation (equal); Methodology (equal); Supervision (equal); Validation (equal); Writing‐original draft (supporting); Writing‐review & editing (equal). **Milan Paunović:** Investigation (equal); Writing‐review & editing (equal). **Milan Plećaš:** Data curation (equal); Investigation (equal); Writing‐review & editing (equal). **Duško Ćirović:** Conceptualization (equal); Funding acquisition (lead); Investigation (equal); Supervision (lead); Validation (equal); Writing‐original draft (supporting); Writing‐review & editing (equal).

## Supporting information

Fig S1

Fig S2

Table S1

Table S2

## Data Availability

All the data used in the analyses are deposited in the Dryad Digital Repository (https://doi.org/10.5061/dryad.cz8w9gj4j).
