# Supplementary figures and images for: Seasonal and diel movement patterns of brown bears in a population in southeastern Europe
Source: Ecol Evol. 2021 Oct 28;11(22):15972–83. doi: 10.1002/ece3.8267 (PMC8601923; doi:10.1002/ece3.8267)

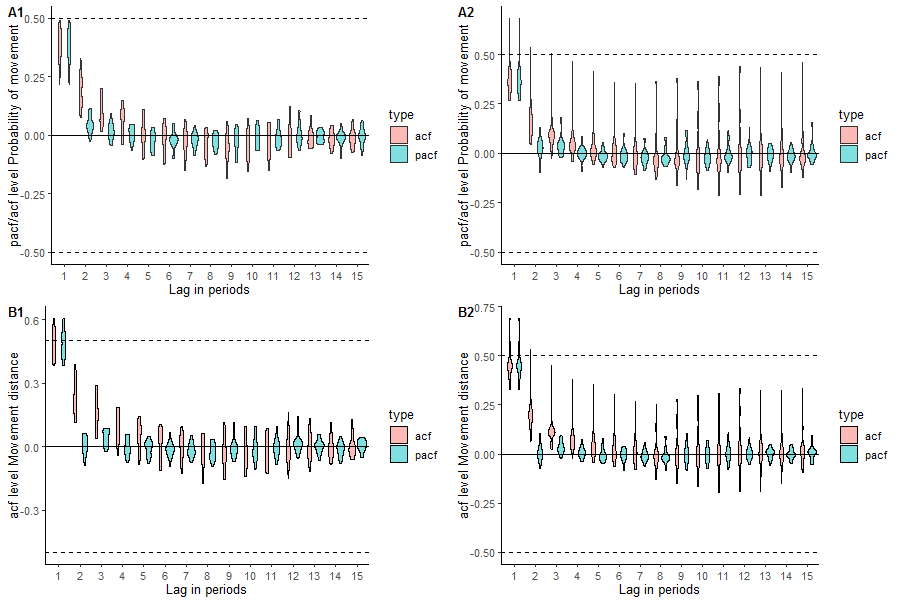

Supplement: Supplementary file 1 — Fig S1 [file ECE3-11-15972-s004.png]

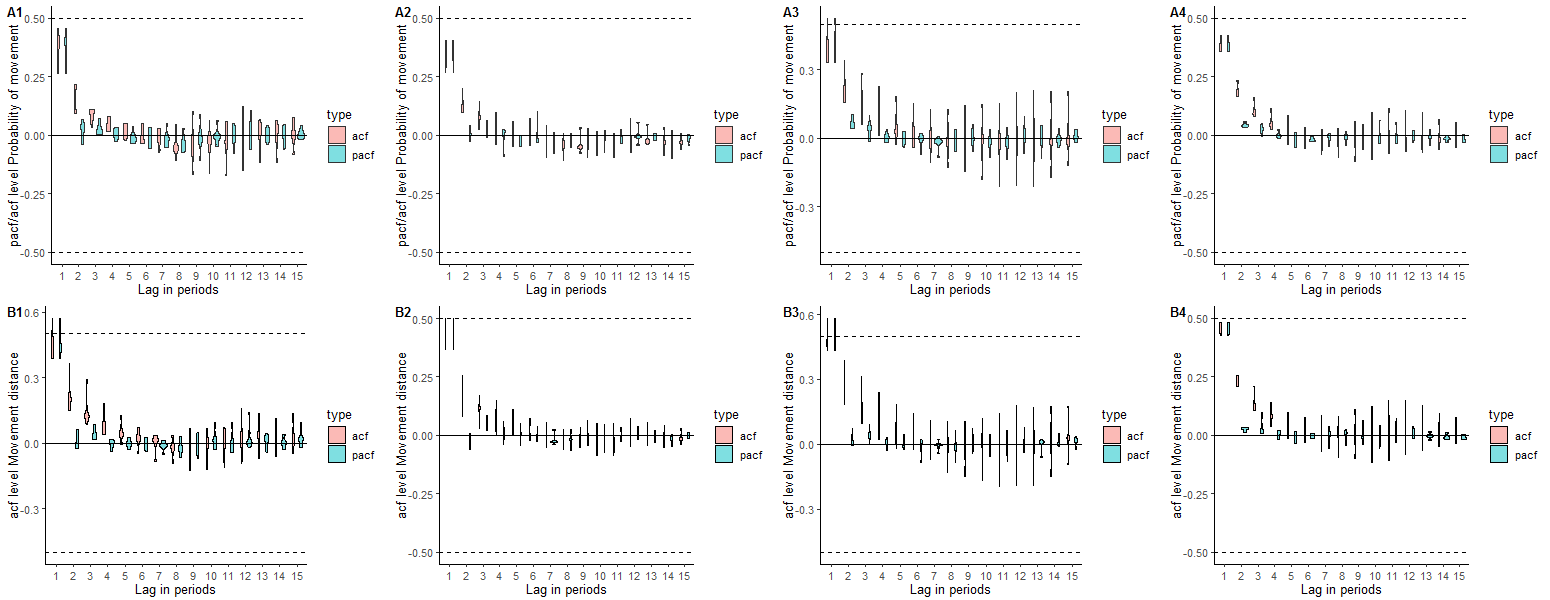

Supplement: Supplementary file 2 — Fig S2 [file ECE3-11-15972-s001.png]
