# Supplementary material for: Seasonal and diel movement patterns of brown bears in a population in southeastern Europe
Source: Ecol Evol. 2021 Oct 28;11(22):15972–83. doi: 10.1002/ece3.8267 (PMC8601923; doi:10.1002/ece3.8267)
Supplement: Supplementary file 3 — Table S1 [file ECE3-11-15972-s002.pdf]

S1 Table. Model selection of candidate models explaining movement distances and probability of movement for four reproductive classes of brown bears (adult male, subadult male, solitary female and female with dependent offspring) in Serbia, 2007-2019. Evaluating metrics include AICc (Akaikes Information Criterion correctedfor small sample sizes), ΔAIC (difference in AICc between the best model (smallest AICc) andeach model) andAICc weight (relative likelihood of a model).

Movement distance		**Mating season (n=18453)**			**Hyperphagia season (n=46189)**		
	df	AICc	ΔAIC	AICcw	AICc	ΔAIC	AICcw
$hour^3$*reproductive classes	10	**71092.6**	**0.00**	**1**	**174900.7**	**0.00**	**1**
$hour^3$	4	72860.1	1767.5	0	175804.7	904.1	0

Probability of movement		**Mating season (n=18453)**			**Hyperphagia season (n=46189)**		
	df	AICc	ΔAIC	AICcw	AICc	ΔAIC	AICcw
$hour^3$*reproductive classes	9	**21655.3**	**0.00**	**1**	**53807.7**	**0.00**	**1**
$hour^3$	3	23045.1	1389.7	0	54389.1	581.4	0
