# Supplementary material for: Seasonal and diel movement patterns of brown bears in a population in southeastern Europe
Source: Ecol Evol. 2021 Oct 28;11(22):15972–83. doi: 10.1002/ece3.8267 (PMC8601923; doi:10.1002/ece3.8267)
Supplement: Supplementary file 4 — Table S2 [file ECE3-11-15972-s003.pdf]

S2 Table. Model selection of candidate models explaining movement distances and probability of movement for four reproductive classes of brown bears (adult male, subadult male, solitary female and female with dependent offspring) in Serbia, 2007-2019. Evaluating metrics include AICc (Akaikes Information Criterion corrected for small sample sizes), ΔAIC (difference in AICc between the best model (smallest AICc) and each model) and AICc weight (relative likelihood of a model).

Movement distance	**Adult males (n=17341)**				**Subadult males (n=12238)**			**Solitary females (n=21473)**		
	df	AICc	ΔAIC	AICcw	AICc	ΔAIC	AICcw	AICc	ΔAIC	AICcw
$hour^{3*}season$	6	**68062.2**	**0.00**	**1**	**47134.2**	**0.00**	**1**	**80741.6**	**0.00**	**1**
$hour^3$	4	68230.6	168.5	0	47416.5	282.3	0	80859.2	117.6	0

Probability of movement	**Adult males (n=17341)**				**Subadult males (n=12238)**			**Solitary females (n=21473)**		
	df	AICc	ΔAIC	AICcw	AICc	ΔAIC	AICcw	AICc	ΔAIC	AICcw
$hour^{3*}season$	5	**21171.5**	**0.00**	**1**	**13046.0**	**0.00**	**1**	**25065.4**	**0.00**	**1**
$hour^3$	3	21357.9	186.5	0	13139.2	93.2	0	25151.0	85.6	0

Movement distance	**Females with offspring (n=13590)**			
	df	AICc	ΔAIC	AICcw
$hour^{3*}season$	6	**50167.1**	**0.00**	**1**
$hour^3$	4	50786.8	619.7	0

Probability of movement	**Females with offspring (n=13590)**			
	df	AICc	ΔAIC	AICcw
$hour^{3*}season$	5	**16343.8**	**0.00**	**1**
$hour^3$	3	16752.4	408.6	0
